# Naturally Occurring Hydroxy Napthoquinones and Their Iron Complexes as Modulators of Radiation Induced Lipid Peroxidation in Synaptosomes

**DOI:** 10.1155/MBD.1997.279

**Published:** 1997

**Authors:** A. S. Kumbhar, S. B. Padhye, Jitender, R. K. Kale

**Affiliations:** 1 Department of Chemistry University of Pune Pune 4110 07 India; 2 Radiation Biology Laboratory School of Life Sciences Jawaharlal Nehru University New Delhi 110 067 India

## Abstract

The modulation of radiation induced lipid peroxidation in synaptosomes by iron (II) and iron (III) complexes of two naturally occurring and therapeutically relevant naphthoquinones viz. 5,hydroxy-1,4 naphthoquinone; juglone and 2,hydroxy-1,4 naphthoquinone; lawsone, have been studied. At lower concentrations the complexes enhance lipid peroxidation predominantly through redox cycling as observed for Fe(II)- juglonate while at higher concentrations the complexes tend to limit lipid peroxidation through fast recombinations.


### NATURALLY OCCURRING HYDROXY NAPTHOQUINONES AND THEIR IRON COMPLEXES AS MODULATORS OF RADIATION INDUCED LIPID PEROXIDATION IN SYNAPTOSOMES

A. S. Kumbhar[1], S. B. Padhye[1]*, Jitender[2], and R. K. Kale[2]

[1] Department of Chemistry, University of Pune, Pune-4110 07, India
[2] Radiation Biology Laboratory, School of Life Sciences, Jawaharlal Nehru University, New Delhi-110 067, India

### Abstract

The modulation of radiation induced lipid peroxidation in synaptosomes by iron (II) and iron (III) complexes of two naturally occurring and therapeutically relevant naphthoquinones viz. 5,hydroxy-1,4 naphthoquinone; juglone and 2,hydroxy-1,4 naphthoquinone; lawsone, have been studied. At lower concentrations the complexes enhance lipid peroxidation predominantly through redox cycling as observed for Fe(II)- juglonate while at higher concentrations the complexes tend to limit lipid peroxidation through fast recombinations.

### Introduction

Lawsone (I) and Juglone (II) are a pair of naturally occurring isomeric hydroxynaphthoquinones which have been cultivated in Africa and India for medicinal and dyeing purposes [1]. The former is the coloring pigment readily extractable from the leaves of *Lawsonia alba* [2] and had been used by the desert travelers as a constituent of mud-plaster used on hands and face to protect against the effects of sun-burns during desert safaris. Juglone is the active principle of *Juglans regia* exuded by its leaves and roots and has been known to exert allelopathic effects [3] on plants growing in the vicinity of the black walnut tree. Both compounds have been employed as radiation modulating agents in Indian Folk medicine but require authentication.

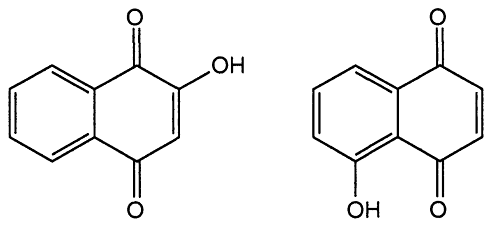

I II

Earlier work in our laboratory had shown that the hydroxyquinones exert most of their biological activities through chelation of trace metals [4]. In this regard complexes of iron and copper were especially found to be biologically active. Since the involvement of iron as the initiator [5] and propagator [6] of lipid peroxidation is known and well established [7] it was thought to be interesting to examine the effect of the low molecular weight ferrous and ferric complexes of I & II on the radiation induced lipid peroxidation in the model system under physiological conditions. Our results indicate that the ferrous complex of II is especially remarkable in enhancing lipid peroxidation through redox cycling while ferric complex of I is also active but independent of redox cycling. These results are relevant for formulating cosmetic materials or therapeutic preparations which include some quantities of I and II.

### Materials and Methods

Trichloroacetic acid (TCA) and thiobarbituric acid (TBA) were from Sigma Chemical Company. All other chemicals and reagents were of analytical grade and were from BDH, Bombay (India). Lawsone was purchased from Sigma Chemical Co. while Juglone was synthesized according to literature method [8], $Fe^{2+}$(lawsone)$_2$($H_2O$)$_2$, $Fe^{3+}$(lawsone)$_3$, $Fe^{2+}$(juglone)$_2$($H_2O$)$_2$ and $Fe^{3+}$(juglone)$_3$ were synthesized according to procedures described earlier [9].

**Preparation of Synaptosomes**

Synaptosomes prepared from the brains of Swiss Albino mice were irradiated with various doses of γ-radiation at the rate of 0.9 Gy/s and the extent of lipid peroxidation was evaluated in terms of malondialdehyde (MDA) formed [10]. To determine the concentration of MDA in the suspension, 1 ml of synaptosomes with or without the drug were transferred into the centrifuge tubes followed by an addition of 1 ml of suspension medium (0.15 M KCl + 10 mM tris HCl), 0.5 ml of 30% trichloroacetic acid (TCA) and 0.5 ml of 52 mM thiobarbituric acid. The tubes were covered with an aluminium foil and placed in the water bath for 30 minutes at 80°C after which they were cooled in an ice bath for 10 minutes and centrifuged at room temperature for 10 minutes. The absorbance of the clear supernatant was measured at 531 nm using a UV 260 Schimadzu spectrophotometer.

**Irradiation**

The irradiation (0.9 Gy/s) of synaptosomes (0.5mg proteins/ml) was carried out in a gamma chamber (5500Ci $^{60}Co$) obtained from Bhabha Atomic Research Center, Bombay (India), at room temperature with the required radiation dose. The dose rate was determined using Fricke's dosimetry. The synaptosomes were used immediately after irradiation for measuring lipid peroxidation.

**Cyclic Voltammetry**

The cyclic voltammetric profiles of the hydroxynaphthoquinones and their iron complexes were recorded as described previously [9].

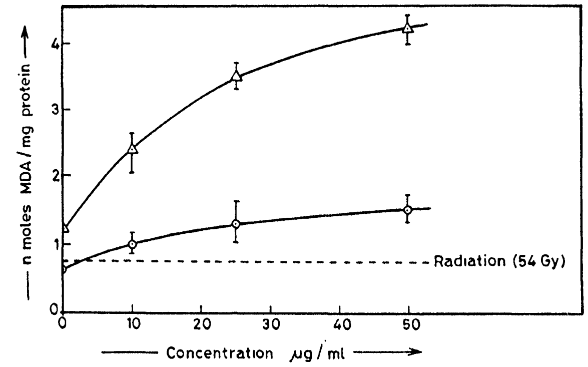

Figure 1. Effect of different concentrations of juglone (O), lawsone (□) on radiation induced lipid peroxidation in synaptosomes. The values represent the mean of 5 experiments ± SD.

**Results and Discussion**

Synaptosomes were irradiated with different doses of radiation (0 to 456 Gy) at a dose rate of 0.9 Gy/s. Lipid peroxidation was found to increase with increase in radiation dose in a sigmoidal manner [11].

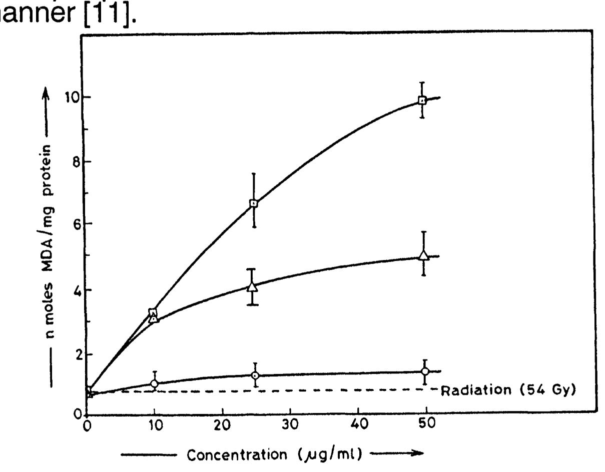

Figure 2. Effect of different concentrations of juglone (O); [$Fe^{2+}$ (juglone)$_2$ ($H_2O$)$_2$] (□); [$Fe^{3+}$ (juglone)$_3$] (Δ) on radiation induced lipid peroxidation in synaptosomes. The values represent the mean of experiments ± SD.

Various concentrations (0-50 μg/ml) of I and II were added to the synaptosome preparations irradiated at 54 Gy. The synaptosomes without these ligands served as the control samples. It is observed that both the ligands enhance lipid peroxidation in a concentration dependent manner (**Figure 1**). The lowered lipid peroxidation in case of II , is probably the result of extremely fast recombination processes of free radicals generated by II due to facilitated redox cycling [12].

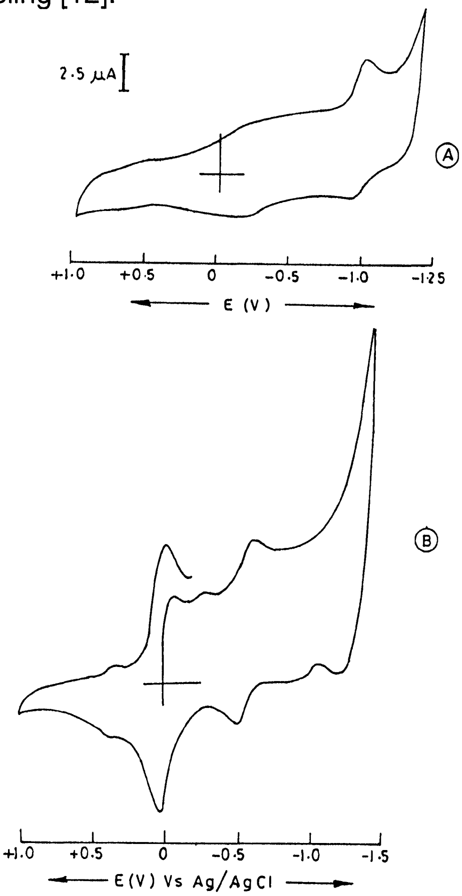

Figure 3. Cyclic voltammograms of 10 mM solutions of (A) [$Fe^{3+}$(lawsone)$_3$] and (B) [$Fe^{3+}$ (juglone)$_3$] in DMSO with TEAP as supporting electrolyte .

A comparison of the modification of lipid peroxidation by iron(II) and iron(III) complexes of II is shown in **Figure 2**. The order of the lipid peroxidation is $Fe^{2+}$(Juglone) > $Fe^{3+}$(Juglone) > Juglone which is in accord with their reduction potentials for the quinone to semiquinone conversions [9].

One plausible mechanism is the oxidation of $Fe^{2+}$ at the expense of dioxygen leading to the formation of superoxide ($O_2^{\cdot-}$ ), hydrogen peroxide ($H_2O_2$) and eventually hydroxyl (OH·) radicals respectively by reactions commonly referred to as the Haber-Weiss and Fenton reactions [13]. The OH· radicals are extremely reactive and initiate lipid peroxidation. It has been shown that the chelating agents can greatly influence the extent to which the above reactions may proceed. Ligands with low affinities for $Fe^{2+}$ do not affect the rate of autooxidation, while the chelators having oxygen donor atoms tend to enhance $Fe^{2+}$ oxidations perhaps due to their greater affinity for $Fe^{3+}$. For example, chelation with EDTA shifts the $Fe^{2+}/Fe^{3+}$ couple potential from -0.77 V to -0.12 V making autooxidation considerably favourable. Similarly the citrate ions are also capable of shifting the $Fe^{2+}$ /$Fe^{3+}$ couple to -0.33 V which leads to a significant enhancement in the rate of $Fe^{2+}$ oxidation. The shift in the $E_{1/2}$ values for the $Fe^{2+}/Fe^{3+}$ couple by some of the chelating agents together with I and II are shown in **Table I**. A comparison of the values indicate that more facile oxidation occurs with these naphthoquinone ligands.

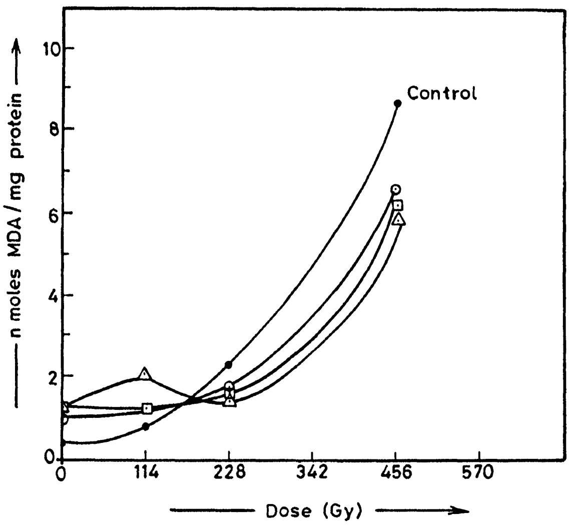

Figure 4. Effect of juglone on radiation induced lipid peroxidation. Synaptosome suspensions (0.5mg/ml) containing different concentrations of juglone 10 µg/ml (O); 25 µg/ml (□); 50 µg/ml (Δ) were irradiated with various doses (0-456 Gy). Values are an average of 5 experiments.

Our cyclic voltammetric studies on the Fe(II) complex of **II** show that the $Fe^{2+}/Fe^{3+}$ redox couple in this complex is observed at the potential of +0.41V [17] while there is no $Fe^{2+}/Fe^{3+}$ couple observed in case of $Fe^{3+}$-(juglone)$_3$ (**Figure 3B**).

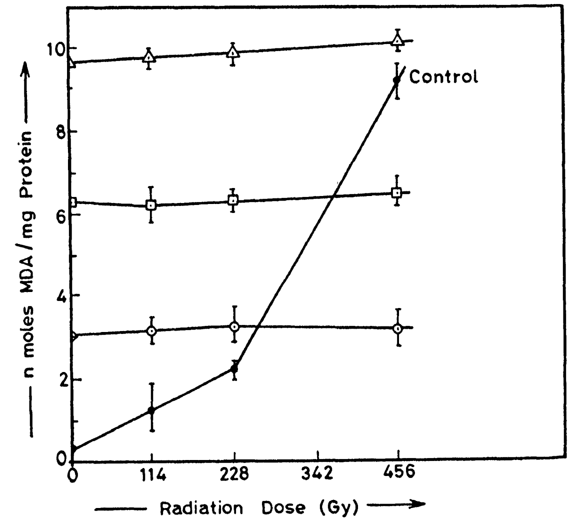

Figure 5. Effect of [$Fe^{2+}$ (juglone)$_2$($H_2O$)$_2$] on radiation induced lipid peroxidation. Synaptosome suspensions (0.5mg/ml) containing different concentrations of [$Fe^{2+}$ (juglone)$_2$($H_2O$)$_2$] 10 µg/ml (O); 25 µg/ml (□); 50 µg/ml (Δ) were irradiated with various doses (0-456 Gy). Values represent an average of 5 experiments ± SD.

This suggests that the oxidation of Fe(II) to Fe(III) should be very facile in the former case which is a pre-requisite for the Fenton type reaction leading to the enhanced lipid peroxidation as observed.

Another possible explanation is that the reduced iron compounds react with the lipid hydroxides (lipid-$O_2$H) to yield alkoxy (lipid-O·) radicals while the oxidised iron compounds yield peroxy radical (lipid-$O_2$·). Both alkoxy and peroxy radicals can stimulate the chain reactions leading finally to lipid peroxidation [13].

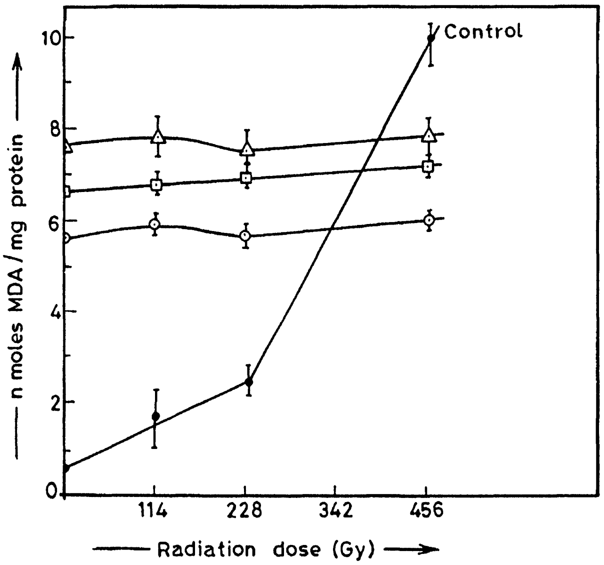

Figure 6. Effect of [$Fe^{3+}$ (juglone)$_3$] on radiation induced lipid peroxidation. Synaptosome suspensions (0.5 mg/ ml ) containing different concentrations of [$Fe^{3+}$ (juglone)$_3$] 10 μg/ml (O); 25 μg/ml (□); 50 μg/ml (Δ) were irradiated with various doses (0-456 Gy).Values represent an average of 5 experiments ± SD.

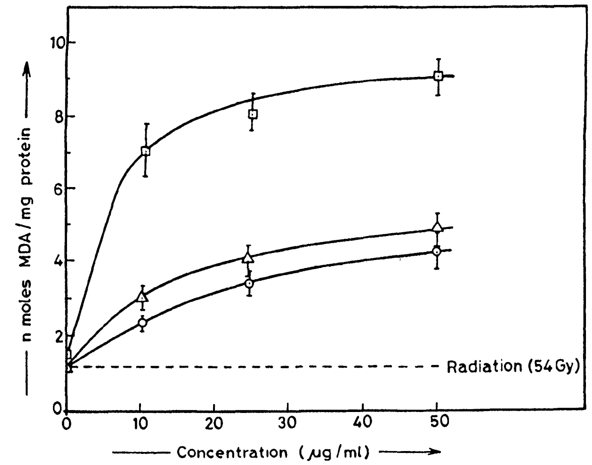

Figure 7. Effect of different concentrations of lawsone (O); $Fe^{2+}$ (lawsone)$_2$($H_2O$)$_2$] (□); [$Fe^{3+}$ (lawsone)$_3$] (Δ) on radiation induced lipid peroxidation in synaptosomes. Values represent an average of of 5 experiments ± SD.

**TABLE I.** The reduction potential for the $Fe^{2+}/Fe^{3+}$ half reaction in presence of ligands

| Ligand | $E_{1/2}$ (V) | References |
|---|---|---|
| $H_2O$ | -0.77 | 14 |
| EDTA | -0.12 | 15 |
| Citrate | -0.33 | 16 |
| Juglone | +0.41 | Present work |
| Lawsone | absent | Present work |

Synaptosome suspensions were also irradiated at the radiation doses of 114, 228 and 456 Gy in the presence of different concentrations (10, 25, 50 μ g/ml) of **II** and its Fe(II) and Fe(III) complexes. It is observed that in the lower dose region (0- 332 Gy) these compounds enhance radiation induced lipid peroxidation while in the higher dose region (452 Gy) these compounds inhibit radiation-induced lipid peroxidation (**Figures 4 to 6**).

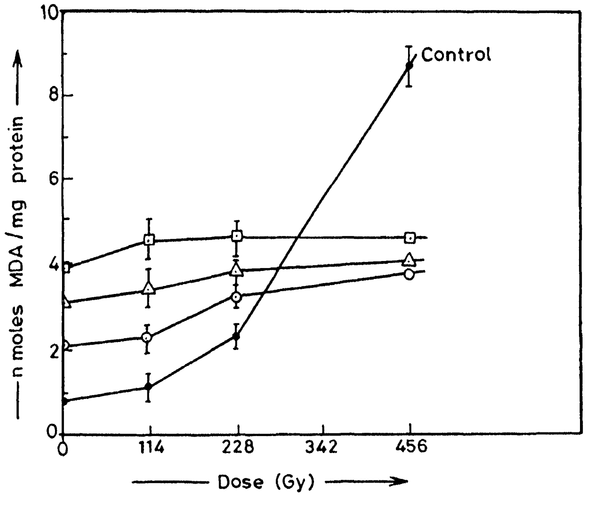

Figure 8. Effect of lawsone on radiation induced lipid peroxidation. Synaptosome suspensions (0.5mg/ml ) containing different concentrations of lawsone 10 µg/ml (O); 25 µg/ml (□); 50 µg/ml (Δ) were irradiated with various doses (0-456 Gy). Values represent an average of 5 experiments ± SD.

The reduction in the lipid peroxidation observed at higher doses is most likely to be due to the promotion of recombination reactions. In another experiment the synaptosomes treated with various concentrations of **I** and its Fe(II) and Fe(III) complexes were examined (**Figure 7**) which revealed a slightly different order of lipid peroxidation as $Fe^{3+}$(Lawsone)$_3$ > $Fe^{2+}$(Lawsone)$_2$ > Lawsone, which indicates that the redox cycling is not a major contributing factor in the lipid peroxidation induced by lawsone derivatives. This is reasonable in view of the quasi-reversible ligand-based redox peaks observed in their electrochemical profiles [9]. Almost all of the observed cyclic voltammetric peaks for **I** and its iron complexes are primarily ligand-based (**Figure 3A**). As observed earlier these compounds also inhibit radiation induced lipid peroxidation at the higher dose levels (**Figure 8 to 10**). Thus lipid peroxidation induced by lawsone and its iron complexes follow a mechanism independent of redox cycling corroborating our observations on their hepatocytes toxicities [9].

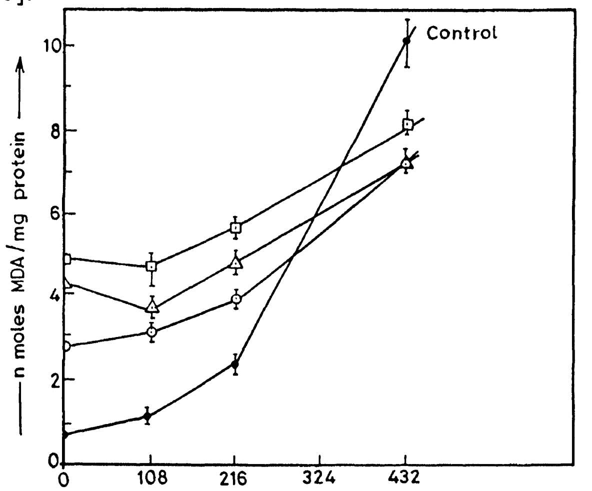

Figure 9. Effect of [$Fe^{2+}$ (lawsone)$_2$($H_2O$)$_2$] on radiation induced lipid peroxidation. Synaptosome suspensions (0.5mg/ml) containing different concentrations of [$Fe^{2+}$ (lawsone)$_2$($H_2O$)$_2$] 10 µ g/ml (O); 25 µg/ml (□); 50 µg/ml (Δ) were irradiated with various dose (0-456 Gy). Values represent an average of 5 experiments ± SD.

The present work has thus shown that two naturally occurring isomeric hydroxynaphthoquinones, viz. lawsone and juglone are capable of modulating lipid peroxidation in synaptosomes either through redox coupling or enzymatically. The radiation protection offered by these compounds at higher doses may be useful for improving radiation therapy of cancer while the radiation damage induced by them at lower concentration needs to be borne in mind when formulating them in the cosmetic or therapeutic preparations.

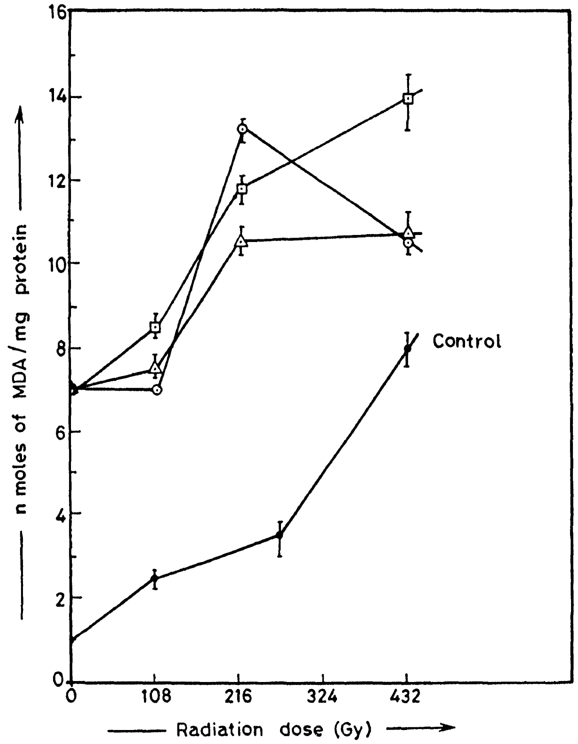

Figure 10.Effect of [$Fe^{3+}$(lawsone)$_3$] on radiation induced lipid peroxidation. Synaptosome suspensions (0.5 mg/ ml ) containing different concentrations of [$Fe^{3+}$ (lawsone)$_3$] 10 μg/ml (O); 25 μ g/ml (□); 50 μg/ml (Δ) were irradiated with various doses (0-456 Gy). Values represent an average of 5 experiments ± SD.

**Acknowledgements**

ASK acknowledges CSIR, New Delhi, for Senior Research Fellowship. SBP would like to thank UGC & CSIR authorities for the support.

**References**

1. Lal B, Dutt S , J. Ind. Chem. Soc., 1933, **10**, 577-579.
2. Brodie AF (1965) in Morton RA (ed.) *Biochemistry of Quinones*, Academic Press , New York , pp. 356-401.
3. Soderquist CJ, J.Chem.Edu., 1973, **50**, 782-783.
4. Padhye S,Kulkarni BA, J.Phys.Chem., 1975, **79**, 927-928.
5. Braughler JM, Duncan LA, Chase RL, J.Biol.Chem., 1986, 261, 10282- 10289.
6. Bucher JR, Tien M, Aust SD, Biochem.Biophys.Res.Commun., 1983, **111**, 777-784.
7. Alper T, Nature, 1968, **217**, 862-863.
8. Jasiatis RG, Krantz A , J.Chem.Ed. ,1972, **49**, 436-437.
9. Kumbhar A, Padhye S, Ross D, Biometals, 1996, **9**, 235-240.
10 .Konnings AWT, Drijver EB, Radiat. Res., 1979, **80**, 494-501
11. Kale RK, Sitasawad SL, Radiat ,Phys, Chem., 1990, **36**, 361-364.
12. Doherty D'Arcy M, Cohen GM, Smith MT, Biochem. Pharmacol., 1984, **33**, 543-549.
13. Halliwell B, Gutteridge JMC, Biochem. J., 1984, **219**, 1-14.
14. Latimer WM (1952) *Oxidative Potentials.*Englewood Cliffs, NJ, Prentice Hall
15. Schwarzenbach G, Heller J, Helv. Chim. Acta., 1951, **34**, 576- 591.
16. Hamm RE, Shull CM, Grant DM, J. Am. Chem .Soc., 1954, **76**, 2111-2114.
17. Garge P, Padhye S, Tuchagues JP, Inorg. Chim. Acta., 1989, **157**, 239-249.

**Received: September 7, 1997 - Accepted: September 24, 1997 - Received in revised camera-ready format: October 16, 1997**

